# Role of Peer Coaching in Transmitting the Benefits of Leader Coaching

**DOI:** 10.3389/fpsyg.2021.679370

**Published:** 2022-01-11

**Authors:** Yanan Dong, Huijuan Dong, Yuan Yuan, Jing Jiang

**Affiliations:** ^1^School of Economics and Management, Beihang University, Beijing, China; ^2^School of Tourism Science, Beijing International Studies University, Beijing, China

**Keywords:** leader coaching, peer coaching, team individualism/collectivism value, team task interdependence, social information processing theory

## Abstract

Drawing on social information processing theory, the present study examines how and when leader coaching can be beneficial for team performance. Based on a sample of 58 teams from a sanitary product company in China, we found that peer coaching served as a mediator linking leader coaching and team performance. Moreover, the team individualistic/collectivism value moderated the first-stage relationship that the relationship between leader coaching and peer coaching was more positive when the team individualism value was low, but not significant when the team individualism value was high; while team task interdependence moderated the second-stage relationship that the relationship between peer coaching and team performance was more positive when the team task interdependence was high, but not significant when it was low. The findings enrich our understandings of the effectiveness of leader coaching behavior by uncovering the theoretical mechanism and boundary conditions. The study also provides important implications for coaching practice in organizations.

## Introduction

Teams have increasingly become the prevalent work unit in the past two decades, due to their ability to effectively respond to dynamic and complex environments faced by organizations (Mathieu et al., 2008; Hu and Liden, 2015). Since leaders are considered as the authority of the teams, with the power to allocate resources and make critical decisions, practitioners and scholars have emphasized the pivotal influence of leaders on team performance (e.g., Porter et al., 2010; Magpili and Pazos, 2018). For example, previous studies have examined the effect of various leadership styles on team performance, such as transformational leadership (Braun et al., 2013), shared leadership (D'Innocenzo et al., 2016), and ethical leadership (Mathieu et al., 2008; Lyubovnikova et al., 2017). However, team success is inseparable from the team members' collective learning and mutual support (Buljac-Samardzic and van Woerkom, 2015), suggesting the important role of learning and developmental-oriented leader behaviors to facilitate these team activities. Leader coaching, which refers to leaders “providing one-on-one feedback and insights aimed at guiding and inspiring improvements in an employee's work performance” (Heslin et al., 2006), has been adopted as a developmental tool to promote team functioning in the workplace (Ellinger et al., 2003; Heslin et al., 2006; She et al., 2019).

Researches have demonstrated that leader coaching is positively related to team performance (Hagen and Gavrilova Aguilar, 2012; Buljac-Samardzic and van Woerkom, 2015). Despite the advantages of leader coaching for team performance, the theoretical mechanisms through which leader coaching is transferred into team performance are underexplored (Liu and Batt, 2010; Buljac-Samardzic and van Woerkom, 2015). Previous research has focused on intermediate team emotional or attitudinal responses (e.g., Weer et al., 2016), however, neglecting the interactions between team members that can leverage the benefits of leader coaching in teams. Teams are complex and dynamic systems that consist of two or more members with different backgrounds or skills (Kozlowski and Bell, 2003; Mathieu et al., 2008). To achieve optimized team outcomes, team members cannot perform in isolation, but they also need to provide feedback, stimulate reflection, and offer psychological support for each other (Lehmann-Willenbrock et al., 2013). Peer coaching, which is defined as the developmental relationship between individuals of equal or similar status to support the professional development of both parties (Bryant, 2005; Wageman et al., 2005; Parker et al., 2008), provides an opportunity to engage in such helping activities in an integrated form (Goldman et al., 2013).

According to the social information processing theory, team members tend to rely on cues from team leaders to understand or interpret what is allowed or encouraged in the team and regulate their cognitional or behavioral responses accordingly (Salancik and Pfeffer, 1978; Shamir et al., 1993). The leader coaching behavior sends the cues or signals so that helping activities are encouraged and team members experience a strong sense of team support, therefore enhancing the team members' commitment to teams and motivating them to engage in more peer coaching activities. Drawing on the social information processing theory (Salancik and Pfeffer, 1978), we propose that leader coaching behavior promotes peer coaching activities, and in turn, enhances team performance.

In addition, research has implied that whether the benefits of leader coaching can transmit to team functioning through peer coaching is contingent on team value and task characteristics (Feldman and Lankau, 2005; Hagen, 2012; Parker et al., 2013). As suggested by social information processing theory (Salancik and Pfeffer, 1978; Shamir et al., 1993), the extent of attention the team members pay to team leaders largely affects their information processing of the leaders' behaviors. Previous research has shown that team individualism/collectivism value could shape the team process (Gundlach et al., 2006) and influence the employees' reactions to leader behaviors (Schaubroeck et al., 2007; Wang et al., 2017). Accordingly, the present study argues that team individualism/collectivism may affect team members' attention to the signals sent by the leader coaching behavior, and in turn, influence the effect of leader coaching on peer coaching. When team members have high individualism value, they tend to be proself-motivated, less cooperative, and unwilling to identify with teams (De Dreu et al., 2008; Wang et al., 2017). Such values prevent team members from paying attention to leader coaching behavior, which indicates the encouragement of helping activities and team support.

Previous research has also suggested that the effectiveness of peer coaching depends on team task characteristics (Parker et al., 2013). The tasks of team members, to more or less extent, are interdependent (Brass, 1985). Task interdependence refers to “the degree to which completing tasks requires the interaction of group members” (Liden et al., 1997). The level of task interdependence determines the need for team members to support each other in pursuit of team-based goals (Shea and Guzzo, 1987; Anand et al., 2018), therefore, may influence the effect of peer coaching on team performance. In all, we propose that team individualism/collectivism serves as the moderator of the first-stage relationship, and team task interdependence moderates the second-stage relationship.

**Figure 1:**
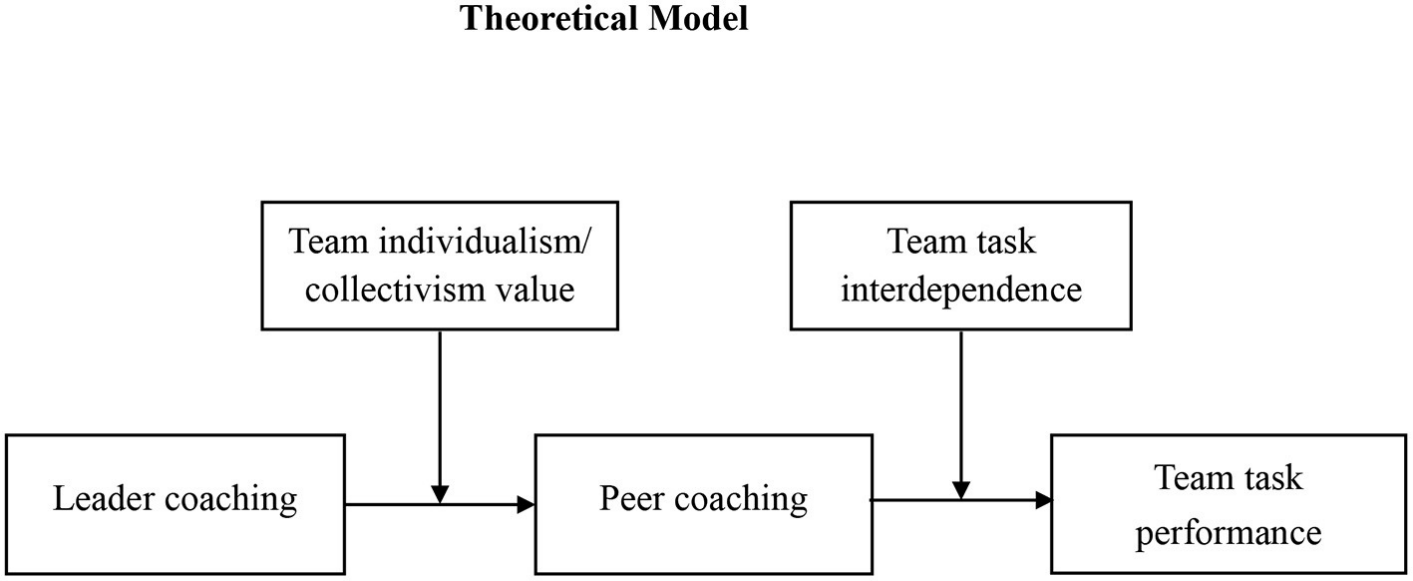
Theoretical model.

The study makes contributions in several ways (the overall model in Figure 1). First, drawing on a social information processing theory, the present research uncovers the mechanism linking leader coaching and team performance from a team interactive perspective, filling in the literature gap about how leader coaching might influence team outcomes (Hagen, 2012; Buljac-Samardzic and van Woerkom, 2015). Second, our research sheds light on the conditions under which leader coaching is more effective by taking team members' values and team task characteristics into consideration, which deepens the understandings of the boundary conditions of leader coaching. Third, most of the peer coaching researches pertains to the field of education (e.g., Huston and Weaver, 2008; Goldman et al., 2013). The present study contributes to the extending literature of peer coaching in management by examining its effectiveness and boundary conditions in the workplace.

## Theory and Hypotheses

### The Mediator of Peer Coaching

Social information processing theory demonstrates that employees develop their cognitions, attitudes, or behaviors as a function of the information available to the team, which often originates from the immediate social environment (Salancik and Pfeffer, 1978). The social environment provides information and cues about what attitudes or behaviors are encouraged, rewarded, and punished (Salancik and Pfeffer, 1978). At the workplace, leaders are one of the main social sources from which team members gather information about the job or interpersonal interaction (Mathieu et al., 2008). Accordingly, confronted with the coaching leadership, team members may process the information suggested by team leaders and correspondingly adapt their behaviors to the leadership environment. Based on the social information processing theory (Salancik and Pfeffer, 1978), we propose that leader coaching can promote peer coaching for the following reasons.

First, employees are likely to look to his or her leaders to learn about the appropriate way to interact with others (Salancik and Pfeffer, 1978; Ambrose et al., 2013; Hu et al., 2018). According to the definition of leader coaching from Heslin et al. (2006), leader coaching consists of three integral components, namely, guidance (the communication of clear performance expectations and constructive feedback regarding performance outcomes, as well as how to improve), facilitation (helping employees to analyze and explore ways to solve problems and enhance their performance), and inspiration (challenging employees to realize and develop their potential). When team members discern frequent coaching behaviors from their leaders, such as providing guidance and feedback, they may regard these activities as an implicit norm or it is encouraged as helping activities, and thus engaging in coaching behaviors toward their teammates. Second, leader coaching behavior can stimulate team members to engage in peer coaching activities through the communication of clear performance expectations. By clearly communicating performance expectations, leaders align employees' goals with team and organizational objectives (Klein et al., 2006; Bennett and Bush, 2014). In such cases, leaders send signals that employees' work is meaningful and has contributions to organizations (Weer et al., 2016). These perceptions enhance their commitment to team tasks and willingness to develop peer coaching relationships (Parker et al., 2013; Weer et al., 2016). Finally, leader coaching behavior involves providing guidance, facilitation, and developing employees' potentials (Heslin et al., 2006). These behaviors convey signals to team members that they are provided with team back-up and support (Rego et al., 2013; Huang and Hsieh, 2015). The interpretation of these signals leads to greater levels of team cohesion and commitment to team members and promotes peer coaching activities (Parker et al., 2008; Rego et al., 2013; Huang and Hsieh, 2015). Facilitating and inspiring coaching behaviors can also promote stronger peer relationships that enable team members to effectively use collective knowledge (Rousseau et al., 2013; Huang and Hsieh, 2015). In all, we propose the following:

H1: Leader coaching is positively related to peer coaching.

Peer coaching is one type of helping relationship that “has the intent of promoting growth, development, maturity, improved functioning” of individuals within it to achieve their job objectives (Parker et al., 2008). Different from other team information processing constructs, such as team task reflexivity, information elaboration, and information sharing, peer coaching emphasizes two-way helping developmental relationships among team members (D'Abate et al., 2003; Parker et al., 2008). Peer coaching involves both interpersonal peer coaching (e.g., providing emotional or psychological support, and resolving potential conflicts among members) and task-related peer coaching (Wageman et al., 2005; Goldman et al., 2013; e.g., giving feedback or advice and developing approaches to solve work-related problems, and enhancing team commitment and shared motivation). In the present study, we propose that peer coaching facilitates team performance for the following three reasons.

First, peer coaching improves team performance since it enables each other to better make use of team members' knowledge (Wageman et al., 2005; Truijen and van Woerkom, 2008). By questioning and providing feedback in the coaching dialogue, coaching peers motivate each other to reflect on one's thoughts or actions, think about what they may not have considered previously, and refine their current working methods (Huston and Weaver, 2008; Parker et al., 2008). In such cases, team members make full use of members' thoughts and knowledge, which in turn help to achieve better team performance (Lyubovnikova et al., 2017).

Second, coaching peers involves providing psychological support for each other and avoiding relational conflicts within teams (Wageman et al., 2005; Huston and Weaver, 2008; Parker et al., 2008; Goldman et al., 2013). The mutual helping relationships and support facilitate the establishment of trust among team members (Halbesleben and Wheeler, 2015). When grounded in trust, team members would be more likely to keep cohesive and commit more to team overall performance (Boies et al., 2015). In addition, peer coaching could increase the collaborations among peers as they tend to build shared motivation and commitment (Wageman et al., 2005). The existing study has provided support for the positive effect of team members' cooperation on team performance (Hu and Liden, 2015).

Third, compared with traditional leader coaching, peer coaching involves non-evaluative opportunities for development as coaching peers tend to be from the same or similar status (Huston and Weaver, 2008; Parker et al., 2008). The non-evaluative relationships provide a relatively safe context for peers to share information or work-related concerns that they will hide from their leaders (Kessel et al., 2012). Previous researches have also provided support that peer coaching can facilitate the flowing of information among team members (Bryant, 2005). When team members engage in open sharing and discussion of information or concerns, they are more likely to come up with better solutions to team task problems and achieve higher team performance (Super et al., 2016). Taken together, we propose the following:

H2: Peer coaching is positively related to team performance.

Combining Hypothesis 1 and Hypothesis 2, we propose that peer coaching mediates the relationship between leader coaching and team performance. Specifically, leader coaching behavior sends the signals, so that helping activities are encouraged in the workplace (Salancik and Pfeffer, 1978). Team members also experience a strong sense of team support and work meaningfulness when receiving coaching from leaders (Rego et al., 2013; Huang and Hsieh, 2015). These perceptions or interpretations motivate team members to engage in peer coaching activities. Peer coaching then enhances team performance through interpersonal and task-related peer helping behaviors (Wageman et al., 2005; Parker et al., 2008). Taken together, we propose the following:

H3: Peer coaching mediates the relationship between leader coaching and team performance.

### The Moderators of Team Individualism/Collectivism Value

Individualism/collectivism is one of the cultural difference dimensions that capture the relative importance of people according to personal interests and shared pursuits (Hofstede, 1980). Individualism reflects the value that personal goals and self-interests are accorded greater importance than collective needs. The opposite of individualism—collectivism—occurs when the needs or interests of teams take precedence over self-interest and personal goals (Hofstede, 1980). Although Hofstede (1980) originally introduced individualism/collectivism at the societal level, many researchers have examined it at lower levels of analysis, such as team level (Schaubroeck et al., 2007). Scholars have argued that teams may develop distinctive cultures (Levine and Moreland, 1991) and values are considered as the defining element of culture (O'Reilly et al., 1991). Team values are defined as the average level of values held by team members (Schaubroeck et al., 2007).

As suggested by social information processing theory, attention paid to the target acts as a key step to process information from the target (Salancik and Pfeffer, 1978). This indicates that whether leader coaching behavior shapes team interactive processes depends on the extent of attention team members pay to the team leader. The present study argues that team individualism/collectivism may affect team members' attention to leader coaching behavior. We propose that team individualism mitigates the positive relationship between leader coaching and peer coaching. Individualism is associated with taking care of themselves and placing a high priority on individual needs and achievements (Hofstede, 1980). Studies have shown that individualists tend to have high proself motivation, be less cooperative, and are unwilling to identify with teams (Gundlach et al., 2006; De Dreu et al., 2008; Wang et al., 2017). In such cases, they have less motivation to pay attention to leader coaching behavior, which indicates the encouragement of helping activities and team support. In addition, leader coaching contradicts the priority needs of personal interests for individualistic team members. The coaching behaviors of leaders foster peer coaching in part by articulating clear expectations for employees' contributions to overall organizational goals (Heslin et al., 2006; Bennett and Bush, 2014), underemphasizing individual interests and achievements. In the peer coaching process where peers provide mutual support and share feedback and advice, individuals' unique contributions to team tasks may be undervalued (Parker et al., 2013). Therefore, individualistic employees are less likely to focus on the coaching behaviors of leaders and be motivated to engage in peer coaching.

In contrast, when individualism is low, that is, team members are high in collectivism, they are more prosocially motivated, cooperative, and tend to engage more in helping activities (Gundlach et al., 2006; De Dreu et al., 2008; Wang et al., 2017). In such conditions, they have high motivation to pay attention to the signal of advocating mutual help and support, which is sent or demonstrated by leader coaching behavior. In addition, collectivists have high needs for affiliation and social relationships (Schaubroeck et al., 2007). They tend to have a stronger attachment to their organizations and subordinate their individual goals to group goals (Earley, 1989). Leader coaching behavior meets these interpersonal needs of collectivistic team members. For example, promoting personal contributions to the organization is compatible with their team-interest priority orientation, and showing support meets the needs for affiliation with the team. Consequently, they are more likely to be motivated by leader coaching to engage in peer coaching. Combining the previous arguments for the positive relationship between peer coaching and team performance (H2), we propose the following:

H4a: Team individualism/collectivism value moderates the positive relationship between leader coaching and peer coaching in such a way that the relationship is stronger when team members have low individualism value rather than high individualism value.

H4b: Team individualism/collectivism value moderates the positive indirect relationship between leader coaching and team performance *via* peer coaching in such a way that the relationship is stronger team members have low individualism value rather than high individualism value.

### The Moderating Role of Task Interdependence

Task interdependence is a team-level phenomenon (Anand et al., 2018), which represents the extent to which team members rely on each other to work on their tasks (Liden et al., 1997; Liu et al., 2017). A high level of task interdependence means that there is a need for team members to coordinate with and assist each other in the pursuit of team goals (Shea and Guzzo, 1987; Anand et al., 2018). In our study, we propose that task interdependence moderates the relationship between peer coaching and team performance in such a way that peer coaching has a more positive effect on team performance when task interdependence is high rather than low.

When task interdependence is high, cooperation among team members is required for team goal accomplishment (Peng et al., 2019). Scholars have demonstrated that peer coaching fosters team cooperation and coordination by providing support and help (Murray et al., 2009), and team cooperation is necessary for increasing team performance (Hu and Liden, 2015). Therefore, peer coaching is more likely to increase team performance in teams with high task interdependence. In addition, high task interdependence increases the usefulness of peer coaching to improving team performance (Baldwin and Ford, 1988). Specifically, peer coaches' advice or guidance is more useful to solve others' problems and improve their performance, since they are more familiar with each other's work when task interdependence is high (Liu and Batt, 2010).

In contrast, when task interdependence is low, peer coaching may not have a positive influence on team performance. Teams with low task interdependence require less cooperation among team members (Peng et al., 2019). As a result, peer coaching, which promotes team members' coordination (Murray et al., 2009), may be considered as not necessary (Liden et al., 1997). In addition, peer coaching, which involves giving guidance and feedback to others, can be interpreted as a deprivation of personal control of independent tasks (Bachrach et al., 2006). For example, when task interdependence is low, these helping behaviors may be construed as feedback of poor performance and negative comments on their working methods (Liden et al., 1997). These interpretations induce team members to feel a sense of threat and regard coworkers' helping behaviors as an encroachment into their personal task domain (Liden et al., 1997), therefore, diminishing the motivation to enhance team performance (Bachrach et al., 2006). Combining the above arguments and the positive relationship between leader coaching and peer coaching (H1), we propose:

H5a: Task interdependence moderates the positive relationship between peer coaching and team performance in such a way that the relationship is stronger when task interdependence is higher.

H5b: Task interdependence moderates the indirect effect of leader coaching on team performance *via* peer coaching in such a way that the indirect positive effect is stronger when task interdependence is high.

### Sample and Procedure

The participants were front-line workers and their immediate leaders from a sanitary product company located in the Guangdong province of China. The task of each team was to produce and improve one of the core products of the company, such as the team room or bathtub. The teams consisted of employees with different expertise or skills related to their core tasks. The present sample is consistent with the aim of the study, as the company has established an apprenticeship system and team members work interdependently to complete the team task. The first author of this article contacted the chief executive officer of the company and secured support for the study. Before our study, we obtained a name roster from the company's human resources (HR) manager and coded our study questionnaires to match leader–employee dyads. For example, the leader in team A was coded as A01, and the employees were coded as A0101, A0102, and A0103... With the assistance of the human resource department, we initially distributed 646 employee questionnaires and 78 leader questionnaires. The participants were required to enter the corresponding codes to access their questionnaires. The codes sent to the leaders and employees at Time 2 were the same as those at Time 1, therefore the survey responses collected at Time 1 and at Time 2 could be matched *via* the corresponding codes. All participation was voluntary. The two types of questionnaires both began with a simple introduction of the research purpose and the assurance of confidentiality. At Time 1, team members were asked to rate their leader's coaching behaviors, team individualistic/collectivist values, and their demographic information. Approximately 1 month later (Time 2), team members were required to evaluate peer coaching and team task interdependence, while team leaders assessed team performance and provided team background information.

After deleting invalid, incomplete, and unmatched cases, 58 teams with 58 leaders and 397 team members constituted the final sample. The response rate was 61.46% for employees and 74.36% for team leaders. The average team size was 6.84 (*SD* = 5.49) and the average team tenure was 6.33 years (*SD* = 5.57). Among the team leaders, 79.31% were male; the average age was 42.44 years (*SD* = 5.74); the average tenure was 9.95 years (*SD* = 4.16), and 72.41% got a bachelor's or higher degree. Among the employees, 77.83% were male, and the majority of them got a college or higher degree (67.00%). The average age and tenure were 38.48 (*SD* = 22.80) and 4.90 (*SD* = 3.65) years, respectively. As for employees' job function, 42.82% worked on manufacturing, 12.85% on administration, 7.56% on customer service, and the remaining worked on research and development, marketing, and others.

### Measures

Responses to all the survey items were provided on a 6-point Likert scale. Since all the items were originally in English, we translated them into Chinese following the translation and back-translation procedures (Brislin, 1980). Unless otherwise noted, the anchors for the items were ranging from strongly disagree (1) to strongly agree (6).

#### Leader Coaching

Leader coaching was measured by Heslin et al. (2006) ten-item scale (α = 0.93), which has been validated in other related research (She et al., 2019). A sample item is “My leader provides guidance regarding performance expectations.” Since the scale contained three sub-dimensions (i.e., guidance, facilitation, and inspiration), we conducted a second-order factor analysis to verify the structure. The results showed that the second-order factor structure fit the data quite well (χ^2^ [31] = 102.18, CFI = 0.97, TLI = 0.96, IFI = 0.97, RMSEA = 0.08, SRMR = 0.03). Thus, following Heslin et al. (2006), we averaged scores across the three sub-dimensions to form an overall measure of leader coaching behavior.

#### Peer Coaching

Peer coaching was measured with an adapted four-item scale (α = 0.93) developed by Wageman et al. (2005), which has been adopted and validated (Dimas et al., 2016). A sample item is “Team members help the team build and use well members' knowledge and skills.”

#### Team Individualism/Collectivism Value

We measured team individualism/collectivism value with a 3-item scale (α = 0.71) developed by Erez and Earley (1987), which has been widely used in relevant research (e.g., Man and Lam, 2003; Schaubroeck et al., 2007). A sample item is “I would rather struggle through a personal problem by myself than discuss it with others.” Higher scores indicate higher individualism and lower collectivism value.

#### Team Task Interdependence

We measured team task interdependence using an adapted three-item scale (α = 0.76) adopted by Liden et al. (1997), the items of which originated from Pearce and Gregersen (1991). A sample item is “We work closely with each other in doing our work.”

#### Team Performance

Zellmer-Bruhn and Gibson (2006) five-item scale (α = 0.96) was used to measure team performance. A sample item is “This team achieves its goals.”

#### Control Variables

We controlled team size because larger teams tend to have more cognitive resources to obtain higher team performance (Hu and Liden, 2015). Team tenure was controlled because the time length of working together may be positively related to team effectiveness (Schaubroeck et al., 2007). We also controlled team function (1 = research and development, 2 = Manufacturing, 3 = Marketing, 4 = Customer service, 5 = Administration, 6 = Others) for its potential effect on team performance.

### Data Aggregation

Because our theoretical model focuses on the team level, we calculated the mean r_wg_ values to capture the degree of agreement among team members on the relevant constructs (James et al., 1984). We also calculated the intraclass correlation coefficients (ICCs) (Bliese, 2000), which indicated the ratio of between-team variance compared with the total variance (ICC1), as well as the reliability of average team perceptions (ICC2). These values were, for leader coaching (*r*_wg_ = 0.76, ICC1 = 0.11, ICC2 = 0.46), peer coaching (*r*_wg_ = 0.86, ICC1 = 0.49, ICC2 = 0.87), team individualistic value (*r*_wg_ = 0.87, ICC1 = 0.59, ICC2 = 0.91), and team task interdependence (*r*_wg_ = 0.93, ICC1 = 0.25, ICC2 = 0.70). The mean *r*_wg_ values were all above the recommended cutoff value of 0.70, indicating acceptable within-team agreement. The cut-off values were above 0.10 (moderate) to 0.25 (strong) for ICC1 scores and above 0.50 (moderate) to 0.60 (strong) for ICC2 scores (LeBreton and Senter, 2008). Although the value of the ICC2 for leader coaching was a little lower than the ideal criteria (0.50), the mean value of *r*_wg_ and ICC1 provide sufficient basis to support the aggregation of the constructs to the team level (Bliese, 2000). Overall, the aggregation of these four variables was justified.

### Analytical Strategy

We examined our theoretical model using Mplus 7.4 (Muthén and Muthén, 2012). We calculated the interaction terms by multiplying the grand-mean centered variables (Aiken and West, 1991). Following previous research (Valls et al., 2016), maximum likelihood with robust standard errors was used for coefficient estimates. The indirect (mediation) and conditional indirect effects (moderated mediation) require the calculation of compound coefficients, which are not normally distrusted. We handed this *via* Monte Carlo simulation procedures (1,000 repetitions) using R (R Development Core Team, New Zealand) to obtain bias-corrected 95% CIs to estimate the indirect effect (Preacher et al., 2010; Preacher and Selig, 2012). This method does not assume the distribution of the product terms (indirect effects and moderation effects) that typically are not normally distributed and yields asymmetric CIs that are faithful to the skewed sampling distributions of the product term. This method has been confirmed to have a higher level of statistical power compared with other methods for the test of indirect effect (MacKinnon et al., 2004).

## Results

### Descriptive Statistics and Correlations

Table 1 showed the means, SDs, and correlations among all the variables in this study. The positive correlation between peer coaching and team performance (*r* = 0.27, *p* < 0.05), as well as leader coaching and peer coaching (*r* = 0.29, *p* < 0.05), provided necessary prerequisites for the analysis among the relevant variables.

**Table 1:** Descriptive statistics and correlations.

**Variables**	**Mean**	**S.D**.	**1**	**2**	**3**	**4**	**5**	**6**	**7**	**8**
1. Team tenure	6.33	5.57								
2. Team size	6.84	5.49	0.28^*^							
3. Team function	3.14	1.67	−0.05	−0.16						
4. Leader coaching	4.93	0.44	0.14	−0.10	−0.07	(0.93)				
5. Peer coaching	4.73	0.61	−0.02	0.06	0.02	0.29^*^	(0.93)			
6. Team performance	4.73	0.89	0.17	0.33^*^	−0.15	0.01	0.27^*^	(0.96)		
7.Team individualism/collectivism value	4.27	0.75	−0.23	0.08	−0.01	−0.21	0.09	0.21	(0.71)	
8. Team task interdependence	5.49	0.33	0.12	−0.04	−0.01	0.23	0.14	−0.10	−0.20	(0.76)

*N = 58*.

* *p < 0.05; Two-tailed test. Team function 1 represents R&D. Team function 2 represents manufacture. Team function 3 represents marketing. Team function 4 represents customer service. Team function 5 represents functional management. Team function 6 represents others*.

### Hypothesis Test Results

Hypotheses 1 predicted the positive relationship between leader coaching and peer coaching. The results in Model 1 of Table 2 indicated that after controlling for team size, tenure and function, leader coaching was positively related to peer coaching (β = 0.30, *p* < 0.01), thus supporting H1. Hypotheses 2 predicted that peer coaching was positively related to team performance. The results in Model 4 of Table 2 showed that peer coaching was positively related to team performance (β = 0.31, *p* < 0.05), even after controlling for leader coaching and team demographic variables. Hence, H2 received support. Hypotheses 3 suggested that peer coaching mediated the relationship between leader coaching and team performance. To test the mediation hypothesis, we produced the 95% CI values by 1,000 resampling using R. The results in Table 3 supported the mediating effect of peer coaching (β = 0.19, bias-corrected 95%CI = [0.01, 0.58], not containing 0). Therefore, H3 was supported.

**Table 2:** Path analytical results.

**Variables**	**Peer coaching team performance**					**Team performance**					
	**Model 1**		**Model 2**			**Model 3**		**Model 4**		**Model 5**	
	**β**	***s.e*.**	**β**	***s.e*.**		**β**	***s.e***	**β**	***s.e***	**β**	***s.e***
**Control variables**											
Team tenure	−0.11	0.13	0.04	0.12		0.15	0.14	0.11	0.15	0.06	0.15
Team size	0.15	0.13	0.05	0.08		0.19	0.14	0.14	0.11	0.17	0.11
Team function	0.07	0.15	0.07	0.13		−0.09	0.13	−0.09	0.09	−0.06	0.10
**Independent variables**											
Leader coaching (LC)	0.30^**^	0.12	0.35^**^	0.09		−0.08	0.13	−0.13	0.17	−0.02	0.18
**Mediators**											
Peer coaching (PC)								0.31^*^	0.13	0.25^*^	0.13
**Moderators**											
Individualism/collectivism value (IC)			0.23	0.11							
Task interdependence (TI)										−0.14	0.12
**Interactions**											
LC^*^IC			−0.23^**^	0.09							
PC^*^TI										0.33^*^	0.13
*R* ^2^	0.13	0.08	0.64^**^	0.08		0.10	0.08	0.15^*^	0.09	0.23^*^	0.09

*N = 58. β represents standardized coefficients. s.e. represents standard error*.

* *p < 0.05*,

** *p < 0.01; Two-tailed test. Team function 1 represents R&D. Team function 2 represents manufacture. Team function 3 represents marketing. Team function 4 represents customer service. Team function 5 represents functional management. Team function 6 represents others*.

**Table 3:** Indirect and moderated indirect results.

**Leader coaching → peer coaching → team performance**			
Moderators		Indirect effect	95%CI
		0.19	[0.01, 0.58]
Team individualism/collectivism	Low	0.34	[0.06, 0.88]
	High	0.11	[−0.03, 0.52]
Team task interdependence	Low	−0.12	[−0.59, 0.17]
	High	0.42	[0.09, 0.95]

**Figure 2:**
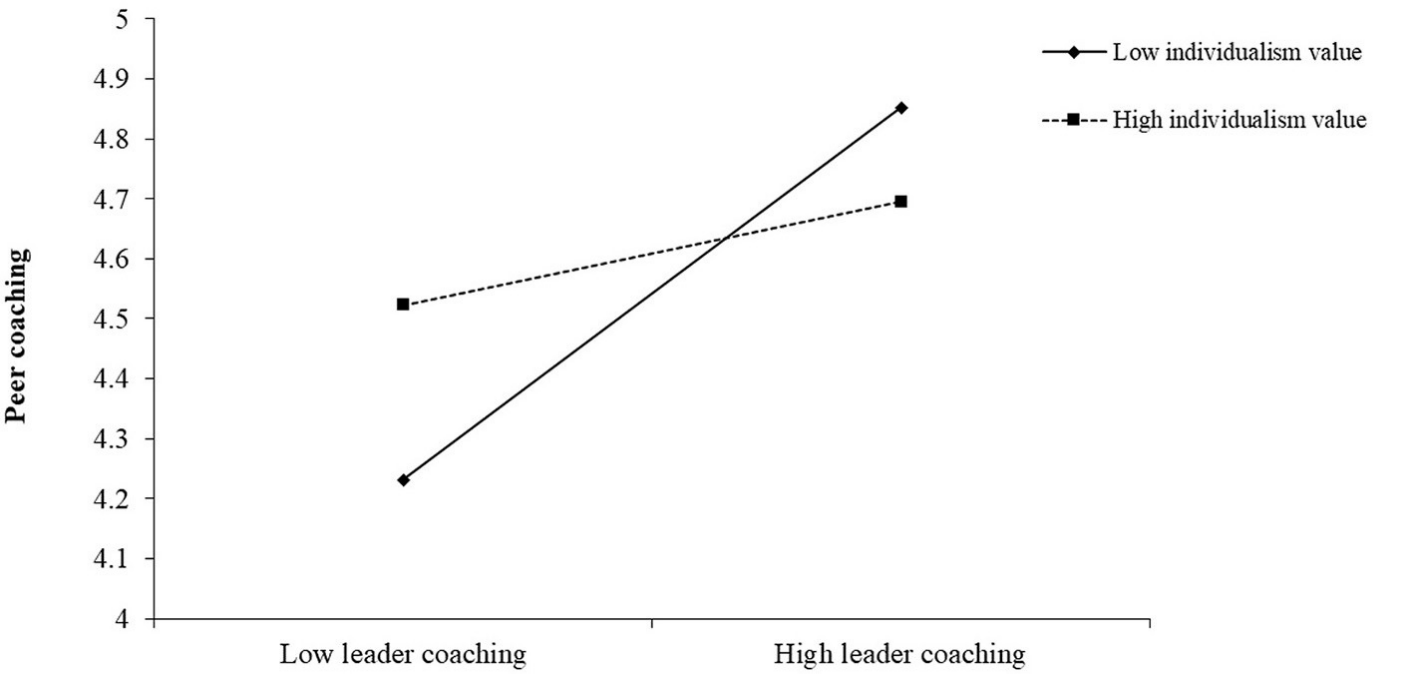
Interactive effect of leader coaching and team individualism/collectivism value on peer coaching.

**Figure 3:**
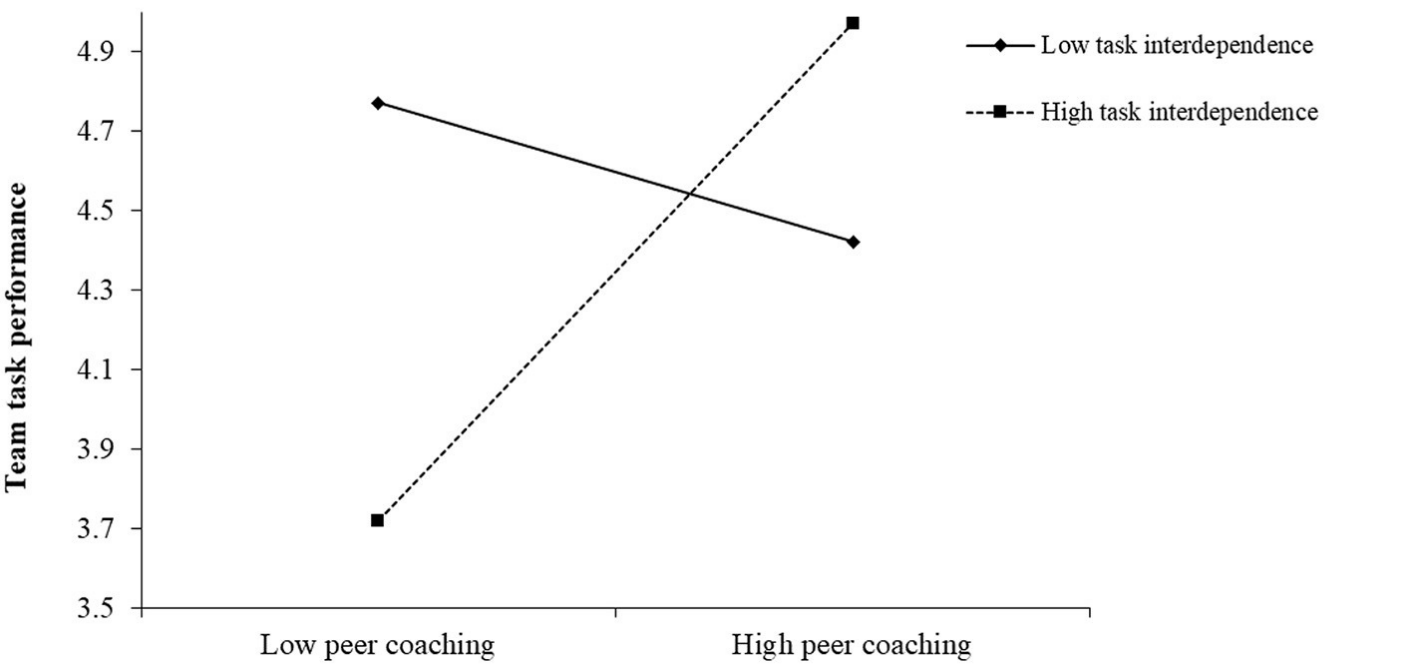
Interactive effect of peer coaching and team task interdependence on team performance.

**Figure 4:**
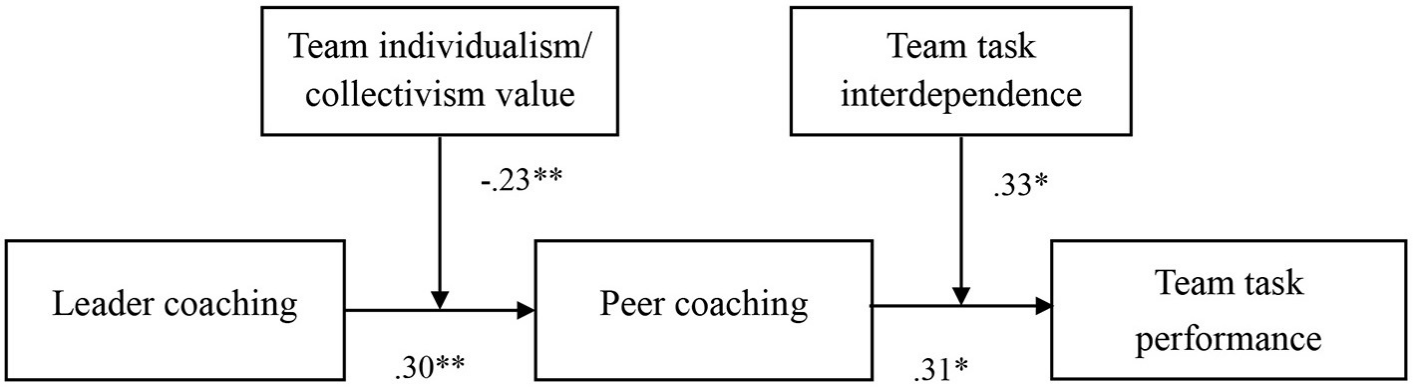
Results of path analysis. *p < 0.05, **p < 0.01.

Hypotheses 4a proposed the moderating effect of team individualism/collectivism value in the relationship between leader coaching and peer coaching. The results in Model 2 of Table 2 supported hypotheses 4a (β = −0.23, *p* < 0.01). Further, we followed Aiken and West's (1991) procedure to plot the relationship between leader coaching and peer coaching at one SD above and below the mean of team individualism value. In Figure 2, the simple slope test suggested that the relationship between leader coaching and peer coaching was significantly positive when the team individualism value was low (β = 0.71, *p* < 0.01), while not significant when the team individualism value was high (β = 0.20, *n.s*.). We followed a similar procedure as Hypotheses 4a to test Hypotheses 5a, which proposed the moderating effect of team task interdependence in the relationship between peer coaching and team performance. The results in Model 5 of Table 2 showed that the interactive effect is significant (β = 0.33, *p* < 0.05). In Figure 3, the simple slope test demonstrated that the relationship between peer coaching and team performance was more positive when team task interdependence was high (β = 1.03, *p* < 0.01), but not significant when it was low (β = −0.29, *n.s*). Hence, both H4a and H5a were supported. The coefficients of all the hypotheses were marked in Figure 4.

Regarding the moderated mediation hypotheses, we tested the conditional indirect effect with the bias-corrected 95% CI value using R (Table 3). Supporting Hypotheses 4b, the indirect relationship between leader coaching and team performance *via* peer coaching was significantly positive when the team individualism value was low (β = 0.34, bias-corrected 95% CI = [0.06, 0.88], excluding 0), while not significant when the team individualism value was high (β = 0.11, bias-corrected 95% CI = [−0.03, 0.52], containing 0). Therefore, H4b was supported. Following a similar procedure, we examined H5b. H5b was also supported by showing that the indirect effect was positive when the team task interdependence was high (β = 0.42, bias-corrected 95%CI = [0.09, 0.95], excluding 0), while not significant when the team task interdependence was low (β = −0.12, bias-corrected 95%CI = [−0.59, 0.17], containing 0).

## Discussion

Based on the social information processing theory (Salancik and Pfeffer, 1978), our study reveals how and when leader coaching behavior improves team performance. Specifically, leader coaching motivates employees to engage in peer coaching, and in turn, enhance team performance. When team members are of high individualism, they are less likely to coach peers under the influence of leader coaching behavior. Team task interdependence acts as another moderator influencing the effect of peer coaching on team performance, in such that peer coaching will lead to higher team performance when team task interdependence is high. We next discuss how this theoretical model and the corresponding findings contribute to theoretical implications and practical insights.

### Theoretical Implications

Our study contributes to the research on leader coaching and peer coaching in the following ways. First, the present study complements the existing research with more empirical evidence for the team-level effect of leader coaching. Although most of the research to date has assumed and verified that leader coaching affects individual-level outcomes, such as employee job satisfaction (Kim et al., 2013), job performance (Huang and Hsieh, 2015), and OCB (Özduran and Tanova, 2017), it is highly believed that leader coaching could also promote team-level outcomes (Hagen and Gavrilova Aguilar, 2012; Rousseau et al., 2013; e.g., Buljac-Samardzic and van Woerkom, 2015). Our research complements the team-level literature by showing that leader coaching can enhance team performance through peer coaching.

Second, drawing on the social information processing theory (Salancik and Pfeffer, 1978), the present study uncovers the mediating role of peer coaching, which enriches our understanding of the underlying theoretical mechanisms of leader coaching. Although previous research has verified the positive effect of leader coaching on team performance (Buljac-Samardzic and van Woerkom, 2015), less attention has been paid to how leader coaching influences team outcomes (Liu and Batt, 2010), especially from a team interactive perspective. In addition, research has suggested that the underlying mechanisms that link leader coaching to individual-level and team-level outcomes might be different (Rousseau et al., 2013; Buljac-Samardzic and van Woerkom, 2015). Leader coaching may impact both individual and team-level outcomes through enhancing the employee and team's skills (Hackman, 2002; Theeboom et al., 2014) and motivation (Pearson, 1991; Geister et al., 2006), or influencing other attitudinal responses. However, the team-level effect of leader coaching could help take a closer look at team interactive mechanisms. Our study provides evidence by showing that leader coaching motivates team members to engage in peer coaching activities, and in turn enhances team performance. The present study answers to scholars' calling for more investigations on how leader coaching might influence team outcomes (Liu and Batt, 2010).

Third, our research contributes to leader coaching literature by unveiling the contingent role of team individualism/collectivism value in transmitting the positive effect of leader coaching on team performance *via* peer coaching. Previous research has examined the positive effect of leader coaching on both team and individual-level outcomes, however, the contexts under which leader coaching is effective lack investigations (e.g., Liu and Batt, 2010; Weer et al., 2016). Our result supports the moderating effect of team individualism/collectivism value by showing that when the team individualism value is high, team members will be less likely to be motivated by leader coaching behavior to engage in peer coaching activities. These results enrich the leadership literature regarding how values affect leadership processes and answer the calling for more research to understand the moderating factors that link leader behaviors to team outcomes (Schaubroeck et al., 2007; Wang et al., 2017).

In addition, the examination of team individualism/collectivism value as a moderator contributes to enriching the boundary conditions of the social information processing theory (Salancik and Pfeffer, 1978). Previous leadership research drawing on the social information processing theory has generally focused on the moderating role of task uncertainty (Ali et al., 2021), leaders' role in repatriate knowledge transfer (Bucher et al., 2020), and team proactive personality (Chiu et al., 2016). Although social information processing theory has implied that the attention employees paid to team leaders could shape the team interactive process (Salancik and Pfeffer, 1978), few studies have directly examined how team value affects the team members' attention and the information processing of leader behaviors. In this vein, our study provides additional empirical evidence of the boundary conditions of social information processing theory.

Finally, our research helps to enrich our understanding of how to integrate a team into a whole to maximize its value, by examining the effect of peer coaching on team performance and the moderating role of team task interdependence. Teams are complex and dynamic systems that consist of two or more members with different backgrounds or skills (Kozlowski and Bell, 2003; Mathieu et al., 2008). Team success largely lies in team members' interactive activities to fully make use of collective knowledge and provide mutual support (Kozlowski and Bell, 2003; Lehmann-Willenbrock et al., 2013). Our results emphasize the important role of peer coaching for team functioning by showing that peer coaching promotes team performance, and team task performance strengthens the benefits of peer coaching on team performance. In addition, although peer coaching has become an important training program in practice to meet the demands of the contemporary business environment (Sue-Chan and Latham, 2004; Parker et al., 2013), few empirical studies have examined its effectiveness in the workplace (Parker et al., 2008). Most of the peer coaching researches pertain to the field of education (e.g., Huston and Weaver, 2008; Goldman et al., 2013). The present study contributes to peer coaching literature in management by providing empirical evidence for its effectiveness in the workplace.

### Practical Implications

Our research has significant implications for practice. First, given the advantages of peer coaching for team performance, organizations should transmit the importance of peer coaching to employees and establish a climate that encourages employees' peer coaching behaviors. Organizations can also make efforts to encourage peer coaching by rewarding employees who participate in these activities. In addition, the positive effect of leader coaching on peer coaching suggests that organizations invest in training programs to improve the leaders' coaching skills. Organizations could also consider hiring leaders who are more willing to coach their employees.

Another implication of our research concerns the contingent role of team individualism/collectivism value and task interdependence. Since team individualism/collectivism values and task interdependence influence the effectiveness of leader coaching behavior, organizations should take into consideration the team members' values and team task characteristics when promoting leader coaching behavior. For example, when the team individualism value is high, they are less likely to be motivated by leader coaching behavior to coach peers. In such conditions, organizations should take additional efforts, such as team-based rewards, to improve their peer coaching willingness (Bamberger and Levi, 2009). Moreover, although the Chinese culture has been widely recognized as collectivistic, it evolves into more individualistic with the infiltration of western culture and young people entering the workplace (Egri and Ralston, 2004). Therefore, it is of great importance to pay attention to employees' value differences when assigning tasks.

### Limitations and Directions for Future Research

Our study has several limitations. First, all data come from the same kinds of sources (e.g., survey data), which might reduce the credibility of our findings. Although our study adopts a multi-source and multi-time design to collect data, it is still difficult to draw causal conclusions from our findings. It is possible that the extent to which team members coach each other determines leaders' coaching behaviors, especially for self-management teams (Morgeson et al., 2010). Therefore, to exclude potential alternative explanations, future research should adopt a quasi-experimental design to explore the causal relationship. In addition, team performance was evaluated by team leaders in the present study, which might be influenced by social desirability bias. However, we suggest that the leader-rated team performance would not interfere with our results for the following reasons. On the one hand, we followed previous research (Schaubroeck et al., 2011; Maruping et al., 2015; Antino et al., 2019) to ask team leaders to rate team performance, as these studies have suggested the relative credibility of team leader-rated performance. On the other hand, to increase the objectivity of leaders' ratings, all the leaders were informed that their responses were confidential and used for academic research only. Nevertheless, we admit that it was necessary to conduct another study with objective team performance or upper-manager-rated team performance in the future.

Second, another limitation concerns the measurements of leader coaching, peer coaching, and team task interdependence used in the present study. Previous research has developed various ways of measuring leader coaching, peer coaching and team task interdependence (e.g., McLean et al., 2005; Williams et al., 2009; Losch et al., 2016). For example, McLean et al. (2005) have developed a coaching scale that emphasizes a team approach to tasks. Wageman et al. (2005) also propose other measurements of leader coaching and team task interdependence. Williams et al. (2009) developed a task-focused peer mentoring scale. In addition, there is debate regarding how individualism and collectivism are measured in the literature. Evidence suggests that they can also be represented as multidimensional constructs. For example, an individual can be both highly collectivist and individualist (Kim et al., 1994). The present study, following previous studies (Gundlach et al., 2006; Schaubroeck et al., 2007), treats these as opposite ends of a bipolar scale along a single dimension. Yet future research could examine whether other measures of these constructs might have different implications for the findings.

Third, our study was conducted only in China, of which the traditional culture is characterized by high collectivism. However, studies have shown that with the global evolution, the culture of western countries is becoming more collectivist while that of eastern Asia is becoming more individualistic (Steele and Lynch, 2013; Ogihara and Uchida, 2014). In consistency with the current tendency, our results demonstrated that the mean value of team individualism/collectivism was 4.27 (in a 6-point Likert scale), suggesting a relatively high level of individualism. The moderating effect of team individualism/collectivism value was supported in the present study in such a way that the positive relationship between leader coaching and peer coaching is stronger when team members have low individualism value rather than high individualism value. By taking into consideration of the cultural values, the findings are more likely to be applied to countries with different cultural backgrounds. However, we do admit that it would be helpful if we could examine our results in western countries with different cultural backgrounds. In addition, our data from only one sanitary product company would limit the generalizability of the present study. In future research, we could examine the hypotheses with a different sample to increase the generalizability.

Fourth, our research does not differentiate leader coaching from other team leaders and compare their potential different effects on team outcomes. Scholars have introduced the concept of team leadership (e.g., Salas et al., 2005; Morgeson et al., 2010), which refers to the leader behaviors serving and directing the team as a whole. Team coaching is a representation of team leadership (Hackman and Wageman, 2005). It emphasizes the role of team leaders in helping members make coordinated and task-appropriate use of their collective resources (Hackman and Wageman, 2005). However, leader coaching, like other traditional leadership models (e.g., transformational leadership or abusive leadership), tends “not to make the distinction between leader–employee interactions and leader–team interactions” (Chen et al., 2007; Eisenbeiss et al., 2008; Morgeson et al., 2010). Leader coaching can have both a one-on-one basis (individual-level) and a collective effect (Matsuo, 2018). Yet future research could investigate more on whether team coaching (team leadership) or leader coaching might have different effects on team outcomes (Morgeson et al., 2010).

Finally, our research does not take into consideration of other antecedents of peer coaching. Previous research has suggested that sharing team activities (e.g., sharing leadership) emerge from specific team relational models or network patterns (DeRue et al., 2015; Wellman, 2017). Given that sharing leadership and peer coaching have some commonalities, future research could benefit a lot by investigating whether certain team relational models or network patterns might lead to peer coaching.

## Conclusion

Enriching our understandings of leader coaching effectiveness, the present study demonstrates that leader coaching promotes team members' peer coaching, and in turn, enhances team performance. In addition, team individualism value mitigates the positive effect of leader coaching on peer coaching, while team task interdependence strengthens the positive effect of peer coaching on team performance. The results highlight a new perspective to explain the effectiveness of leader coaching and provide insights for organizational management practices.

## Data Availability Statement

The original contributions presented in the study are included in the article/Supplementary Material, further inquiries can be directed to the corresponding author/s.

## Ethics Statement

The studies involving human participants were reviewed and approved by Tsinghua University, China. The patients/participants provided their written informed consent to participate in this study.

## Author Contributions

YD made statistical analysis and wrote the theoretical session of paper. JJ wrote the results session and refine the whole manuscript. HD and YY revised and edited the paper.

## Conflict of Interest

The authors declare that the research was conducted in the absence of any commercial or financial relationships that could be construed as a potential conflict of interest.

## Publisher's Note

All claims expressed in this article are solely those of the authors and do not necessarily represent those of their affiliated organizations, or those of the publisher, the editors and the reviewers. Any product that may be evaluated in this article, or claim that may be made by its manufacturer, is not guaranteed or endorsed by the publisher.
